# Development of a Comprehensive Food Literacy Measurement Tool Integrating the Food System and Sustainability

**DOI:** 10.3390/nu12113300

**Published:** 2020-10-28

**Authors:** Dahyun Park, Yoo Kyoung Park, Clara Yongjoo Park, Mi-Kyung Choi, Min-Jeong Shin

**Affiliations:** 1 Interdisciplinary Program in Precision Public Health, Graduate School, Korea University, Seoul 02841, Korea; ekgus7171@korea.ac.kr; 2 Department of Medical Nutrition, Graduate School of East-West Medical Science, Kyung Hee University, Yongin 17104, Korea; ypark@khu.ac.kr; 3 Department of Food and Nutrition, Chonnam National University, Gwangju 61186, Korea; parkcy@jnu.ac.kr; 4 Faculty of Food and Health Sciences, College of Natural Sciences, Keimyung University, Daegu 42601, Korea; 5 Department of Biosystems and Biomedical Sciences, College of Health Science, Korea University, Seoul 02841, Korea

**Keywords:** food literacy, questionnaire, eating behavior, food consumption, health literacy, health education, food chain, sustainable development

## Abstract

The concept of food literacy is evolving and expanding, increasing the need for a comprehensive measurement tool for food literacy. This study aimed to develop a validated food literacy questionnaire based on an expanded conceptual framework for food literacy. A literature review of existing frameworks and questionnaires for food literacy and focus group interviews (*n* = 12) were conducted to develop a conceptual framework and candidate questions. A Delphi study (*n* = 15) and pilot survey (*n* = 10) to test the preliminary questionnaire’s content and face validity were conducted, which were followed by the main survey (*n* = 200). Construct validity and reliability were assessed using exploratory factor analysis (EFA) and Cronbach’s alpha, respectively. Criterion validity was assessed by comparing food literacy scores with food knowledge scores (FN-score) and nutrient quotient scores (NQ-score). By integrating and revising the six existing conceptual frameworks and focus group interview results, we proposed a two-dimensional conceptual framework comprising a literacy dimension and a food system dimension. After reviewing 560 items and categorizing them into 18 domains (3 in the literacy dimension × 6 in the food system dimension), 32 questions were selected. As a result of the Delphi study, two items were deleted, and content validity was confirmed for the remaining 30 items (content validity ratio (CVR) = 0.92). Ten items were revised during the face validation process, and five items were excluded as a result of the EFA. The final food literacy questionnaire comprised 25 questions related to five factors: production, selection, preparation and cooking, intake, and disposal. Food literacy scores were positively associated with FN- and NQ-scores, confirming the reliability and criterion validity of the final questionnaire. The two-dimensional food literacy conceptual framework developed in this study systematically encompasses complex food literacy concepts by adding a food systems dimension (production, selection, preparation and cooking, intake, and disposal domain) to the existing literacy dimension (functional, interactive, and critical literacy domain). Based on this integrated conceptual framework, a 25-item food literacy questionnaire was developed and validated for practical use.

## 1. Introduction

Poor dietary intake is a major risk factor for non-infectious diseases, as such, 11 million deaths and 255 million disability-adjusted life years globally are attributed to dietary risk factors annually [1]. Additionally, more than half of the 17 goals of the Sustainable Development Goals (SDGs) of the United Nations are related to food and nutrition, such as food security, health, consumption and production patterns, and the environment [2]. Currently, the importance of diet and the food system are expanding into various areas in addition to personal health. In addition, relationships between the food system and individuals have been complicated and rapidly evolving [3,4]. Therefore, to understand the importance of diet and support health promotion and sustainability of the environment, economy, and society, individuals must have the capacity to explore and understand food and the complex food system.

Food literacy has been described by Vidgen and Gallegos as “the scaffolding that empowers individuals, households, communities or nations to protect diet quality through change and strengthen dietary resilience over time. It is composed of a collection of inter-related knowledge, skills and behaviors required to plan, manage, select, prepare and eat food to meet needs and determine intake [5]”. Food literacy differs from food knowledge (knowledge on agri-food certification, nutrition labeling, food safety, etc.) as food knowledge alone cannot benefit an individual’s health or the society. Thus, food literacy may benefit dietary behaviors by helping individuals understand the concept of healthy and sustainable diet and how to choose more appropriate foods. The concept of food literacy has been derived from health literacy, which is positively associated with healthy dietary habits [6,7,8] and may affect health conditions, such as obesity [9,10]. However, research is limited on the association between food literacy and dietary intake and habits. While the assessment of food literacy is crucial as it provides a basis for establishing strategies to improve food literacy and measure its performance [11], existing food literacy measurement tools have some limitations. First, the existing tools mostly focus on nutrition-related health literacy. However, as the role of food goes beyond personal health, it is necessary to encompass the social, economic, and environmental effects of food literacy. Although some food literacy researchers has incorporated this aspect [12,13,14], a more systemic approach is needed to measure food literacy at the individual and society levels in a balanced manner [15]. Moreover, the existing tools were developed and validated in Western populations, pointing to a need for food literacy measurement tools for individuals with other cultural backgrounds. Therefore, this study aimed to establish a comprehensive conceptual framework for food literacy, and thereby develop a validated measurement tool based on this framework applicable to the Korean population.

## 2. Materials and Methods

The study design consists of three steps. First, a conceptual framework was established through a literature review and qualitative research. Next, candidate questions were derived based on the literature review. Through a Delphi survey and pilot survey of the candidate questions, content and face validity were verified, respectively, and the questions were revised accordingly. Lastly, to finalize the questionnaire, a survey was conducted to assess construct validity and reliability. All processes in this study were approved in advance by the Institutional Review Board of Korea University (KUIRB-2019-0306-01). All study participants voluntarily consented to participate in the study.

### 2.1. Conceptual Framework Development

Prior to developing the food literacy questionnaire, we conducted a literature review to examine the existing conceptual framework related to food literacy. We then assessed how the literacy dimensions derived from the literature review were perceived by the general public through a focus group interview (qualitative study). Conceptual content within each domain of two dimensions was reorganized according to the interview results. Then, a conceptual framework was developed by integrating the literature review and qualitative research results.

#### 2.1.1. Literature Review

A literature search was conducted on studies published within five years, as of 24 June 2020, through PubMed, Web of Science, RISS, and KISS. Titles were searched using the key term “Food literacy.” Inclusion criteria for papers were as follows: (1) included a conceptual framework, (2) written in either English or Korean, and (3) had an accessible full text.

#### 2.1.2. Qualitative Study

To confirm the conceptual content of food literacy, we conducted focus group interviews with adults aged 20–64 years recruited by poster and internet postings in an urban university and a rural community service center. As differences in access to and understanding of food information may exist depending on the residential area [16,17], interviews were performed with two groups according to whether participants resided in an urban or rural area. The ideal number of participants per group was determined to be six [18,19]. The interview questions are shown in Table 1. All interviews were recorded and transcribed. After transcription of the interview, the internal structure of the phenomenon being studied was analyzed by coding. This involves systematizing the data, by investigating the vocabulary, phrases, subjects, and scenes that repeatedly appear in the data and assigning a certain code. After coding, classification criteria were set for each subject, and materials with similar contents were reclassified [20]. Three researchers independently analyzed the transcripts. Finally, the analysis was completed through a category identification process.

### 2.2. Development of a Preliminary Questionnaire

#### 2.2.1. Candidate Item Development

A question pool was created from the existing food literacy questionnaires identified through the literature review. Questions that appeared with high frequency in previous food literacy questionnaires were primarily selected and categorized according to the developed conceptual framework. For domains with missing questions, new questions were developed based on the conceptual content identified in the focus group interviews. The previously and newly developed questions were then reviewed and revised according to the following criteria: (1) questions were removed if they measured characteristics limited to a specific age group or gender, (2) if the meaning of one item overlapped with the meaning of another, one was removed; and (3) if multiple questions measured the same domain, the question that elicited a more detailed response or was easier to understand was retained, and other items were removed.

#### 2.2.2. Delphi Study

To confirm the candidate questionnaire’s content validity, a Delphi survey was conducted in 15 experts with professional experience in the field of food and nutrition recommended by relevant academic societies(The Korean society of clinical nutrition, etc.). To calculate the content validity ratio (CVR), the validity of each item was assessed on a four-point Likert scale, as follows: very inappropriate, inappropriate, appropriate, and very appropriate. CVR was calculated as CVR = $\frac{N_{e}-\frac{N}{2}}{\frac{N}{2}}$ [21,22]. Ne is the number of experts who rated a question as valid (i.e., appropriate or very appropriate), and N is the total number of experts. Content validity was considered appropriate when CVR was 0.49 or higher [21]. We requested suggested revisions for inappropriate or very inappropriate questions. The questions were thus revised, and the survey was repeated until all questions were determined to have appropriate validity.

#### 2.2.3. Pilot Survey

A pilot survey was conducted in the general population to assess the face validity of the revised candidate questionnaire. Ten adults aged 20–64 years were recruited through posters and online recruitment notices in two universities located in urban areas through convenience sampling. We used interviews to investigate whether the questions were ambiguous or too complex.

#### 2.2.4. Preliminary Questionnaire

The preliminary questionnaire comprised questions on food literacy, food knowledge, diet quality, and individual characteristics. The 25 food literacy questions were designed to allow the respondent to assess his or her own abilities using a 5-point Likert scale, as follows: strongly disagree = 1 point, disagree = 2 points, neutral = 3 points, agree = 4 points, and strongly agree = 5 points. Food literacy score was calculated by summing all responses. Other questions were provided to assess the association between food literacy and food knowledge or diet quality. The level of food knowledge was evaluated using a food knowledge score (FN-score) questionnaire developed by three of the study researchers (D.P., M.-K.C., and M.-J.S.) comprising six questions which were related to agri-food certification, expiration date, nutrition labeling, and food safety. The FN-score is the number of questions correctly answered, ranging from 0 to 6. Diet quality was assessed using the nutrition quotient-score (NQ-score). A nutrition quotient (NQ) was developed by The Korean Nutrition Society to assess the overall dietary quality and food behaviors of Korean adults [23]. The NQ-score is calculated by a 21-item checklist composed of questions related to nutrition balance, food diversity, moderation of amount of food intake, and dietary behavior. A higher NQ-score indicates better diet quality and food behavior.

### 2.3. Validation of the Final Questionnaire

#### Main Survey

Using the preliminary questionnaire, the main survey was conducted in August 2020, with 200 Korean adults aged 19–64 years, either online or offline, through convenience sampling. The appropriate sample size was determined according to the following criteria: (1) in the case of low factor loading (0.4), more than 200 samples would be required [24]; and (2) samples were required to be approximately five times the number of questions [25]. To maximize sample heterogeneity, gender and age were balanced, and residential area was sampled to resemble the population distribution of Korea [26].

### 2.4. Statistical Analysis

Exploratory factor analysis (EFA) using principal component analysis with varimax rotation was conducted to verify construct validity, thus determining the number and nature of underlying factors of the questionnaire, based on the main survey results. The Kaiser–Meyer–Olkin (KMO) method was used to measure sample appropriateness. Bartlett’s test of sphericity and total variance explained were used to evaluate the factor analysis. According to the EFA results, each item was assigned to the factor with the highest factor loading. Questions that did not meet the criteria (i.e., communalities extraction < 0.4, factor loading < 0.4) were deleted. Cronbach’s alpha was calculated to confirm internal consistency for the total questionnaire and each factor. A Cronbach’s alpha value exceeding 0.7 was considered to indicate reliability [27]. A criterion validity analysis was conducted to confirm the association of food literacy scores with FN- or NQ-scores. Linear regression analyses were used. Model 1 indicated the crude analysis, while Model 2 additionally adjusted for age, gender, and household income. All analyses were conducted using the Statistical Package for Social Sciences for Windows, version 24.0. IBM Corp: New York, United States.

## 3. Results

### 3.1. Conceptual Framework Development

#### 3.1.1. Literature Review

During the literature review, we found 125 studies containing “food literacy” in the title. Of these, six described a conceptual framework for food literacy [5,12,13,14,28,29]. Table 2 summarizes the food literacy conceptual framework derived from the literature review. The six existing food literacy conceptual frameworks comprised various components, making it difficult to use a single criterion for classification. Four consisted of food-related components, such as food and nutrition knowledge [5,12,13,29], while the other two included components on abilities related to processing food-related information, such as functional competency [14,28].

**Figure 1:**
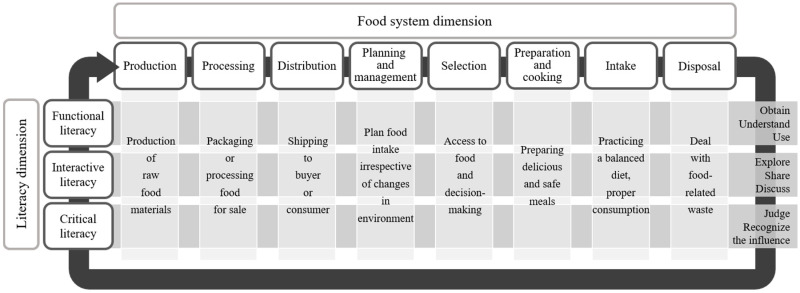
Food literacy conceptual framework derived from the literature review.

To integrate this wide variety of components, we proposed a two-dimensional conceptual framework (Figure 1). The first dimension integrated various components of information processing abilities related to food literacy. Previous frameworks indicated skills, behaviors, knowledge, and decisions [13,29] regarding food literacy, which can be covered by Nutbeam’s theory, the most comprehensively and widely used model [14,28] originally derived for health literacy. Thus, this literacy dimension was composed of three domains, according to Nutbeam’s model: functional literacy, interactive literacy, and critical literacy. The second dimension integrated components related to the food system. Vidgen and Gallegos’s classification system, consisting of planning and management, selection, preparation, and intake, is the most commonly used; however, it does not encompass broader functions of food or sustainability for the environment and society. Other studies have used theoretical concepts such as ecologic (beyond the self), self-efficacy and confidence, and community food security [12,13,29]. Therefore, to integrate these components and fully embrace sustainability for the environment and society, the second dimension consisted of eight domains of the food system: production, processing, distribution, planning and management, selection, preparation and cooking, intake, and disposal. These domains are synthesized by prior studies combined by food system domains [30]. The two-dimensional conceptual framework is shown in Figure 1. The food system dimension has eight domains including production, processing, distribution, planning and management, selection, preparation and cooking, intake, and disposal. Further, the literacy dimension has three domains including functional, interactive, and critical literacy. Each domain contains various components, as shown below. The two-dimensional model means that the concept of food literacy is subdivided into 24 areas by crossing the eight domains of the food system dimension and three domains of the literacy dimension.

#### 3.1.2. Focus Group Interviews

The 12 focus group participants (*n* = 6 per group) were evenly distributed by gender, age, and location of residence. The participants included 6 men and 6 women aged 24–54 years (mean: 35.6 ± 9.3 years). Of these participants, six (50%) lived in urban areas and the others lived in rural areas. Interviews lasted for approximately one hour. During the interviews, various components of food literacy were derived and categorized based on the conceptual domains of food literacy identified in the literature review (Table 3). Among the eight conceptual domains, most were composed of several elements. Only the “planning and management” domain consisted of a single element: “price and time.” However, during the focus group interviews, this was mainly discussed within the context of selecting foods, rather than planning menus or managing a food budget. Therefore, this element was re-categorized to the “selection” domain, and the “planning and management” domain was removed.

### 3.2. Development of a Preliminary Questionnaire

#### 3.2.1. Candidate Item Development

The 547 questions of previous food literacy questionnaires identified through the literature review (see Supplementary Table S1) were classified into 21 areas (three food literacy domains × seven food system domains), based on the conceptual framework. Thirteen new questions were developed for empty components or missing essential questions using keywords derived from the focus group interview. Based on the exclusion criteria mentioned in the Method section, 528 items were deleted from the question pool (mostly due to the redundancy of the questions), and 32 items were identified as candidate questions.

#### 3.2.2. Delphi Study

Through the Delphi study, the questions were revised according to expert feedback, and the content validity of the 32 questions was confirmed through CVR. The Delphi study was conducted three times until consensus was reached. During this process, two items were deleted. The average CVR value of the remaining 30 items was 0.92. By the feedback of experts, the items including the “processing” domain were reclassified into the “selection” domain, which was approved by all experts. This means information about “processing” is meaningful to general consumers only in the context of “selection”. Consequently, the preliminary questionnaire consists of six food system domains. The total numbers of items corresponding to the production, distribution, selection, preparation and cooking, intake, and disposal domains of the food system dimension were five, three, eight, five, six, and three, respectively. Additionally, there were 16, 7, and 7 items corresponding to the functional, interactive, and critical literacy domains of the literacy dimension, respectively.

#### 3.2.3. Pilot Survey

Through the pilot survey to confirm face validity, most items were found to be easy to understand for the general public and suitable to evaluate food literacy. Some questions were partially revised to clarify their meaning: six questions were rewritten with simpler or more specific vocabulary, and examples were added to four questions to enhance understanding.

### 3.3. Validation of the Final Questionnaire

#### Main Survey to Test Validity

In total, 200 respondents participated in the main survey (online: *n* = 138, offline: *n* = 62). Participant characteristics are shown in Table 4. Participants were evenly distributed with respect to gender, age group, household income, and education, while the distribution of place of residence was representative of the Korean population.

The preliminary questionnaire’s validity was confirmed by EFA using Principal component analysis estimation (Table 5). Two items with communalities of 0.4 or less, and three items with factor loading values less than 0.4, were excluded. Five factors were derived from the 25 items included in the final analysis: preparation and cooking (Factor 1), production (Factor 2), selection (Factor 3), disposal (Factor 4), and intake (Factor 5). The KMO measure of sampling adequacy was at an appropriate level (0.899), and the Bartlett’s test result was statistically significant (X^2^ = 2396.744, df = 300, and *p* < 0.001). As a result of the reliability analysis, the Cronbach’s alphas for each of the five factors were all above 0.7: Factor 1 = 0.843, Factor 2 = 0.871, Factor 3 = 0.770, Factor 4 = 0.763, and Factor 5 = 0.703. Cronbach’s alpha for the total questionnaire was 0.928. The results were similar to those using maximum likelihood (ML) estimation and oblimin rotation (data not shown).

Food literacy scores were positively associated with having both objective food knowledge and good diet quality as measured by FN- and NQ-scores, respectively (Table 6). As the FN- and NQ-scores increased by one unit, food literacy scores increased by 0.028 and 0.351, respectively. The statistical significance of the two associations did not disappear after adjusting for age group, gender, and household income level.

## 4. Discussion

In this study, a validated food literacy measurement tool was developed based on a two-dimensional conceptual framework comprising a literacy dimension and a food system dimension. In addition, a 25-item food literacy questionnaire for adults was developed and validated based on this integrated conceptual framework. The results are important in that our conceptual framework expands the concept of food literacy to the food system, and thereby to sustainability for the environment and society as well.

Accumulating studies have recognized food literacy as a branch of health literacy [14]. The currently most cited definition of food literacy proposed by Vidgen and Gallegos [5] as quoted in the introduction is meaningful, as it embraces the elastic and dynamic components of food literacy in response to an ever-changing and complex food environment [31]. Recently, interest in the complex environment and social components related to food has increased, and the concept of food literacy has expanded beyond its association with human health [12,13,29,32]. Indeed, the Food and Agriculture Organization of the United Nations has emphasized that the food system must be considered within the context of rapid worldwide population growth and urbanization, changes in consumption habits, globalization, and climate change [33]. However, no studies to date have suggested a conceptual framework that could encompass this flow of food literacy research and food system. Our study, by integrating the food system into a conceptual framework of food literacy, may overcome the limitations of previous approaches in solving nutritional problems such as food safety, nutrition, and sustainable diet [33]. In addition, this study is distinct in that we included a general population sample at an average of 38.6 years old (ranged from 19–63 years) when developing the conceptual framework and assessing the measurement tool’s validity. Unlike the previous study [5] conducted in participants only aged 16–25 years, our results in the focus group interviews showed that the elements of planning and management were reclassified to the selection domain. It can be speculated that they were conceptualized as immediate reactions at the time of food selection and consumption, rather than when planning and managing a dietary plan. This suggests that although theoretically planning and managing and selecting are components of two different food system domains, in practice, these domains may appear as a single action.

In the present study, a 25-item food literacy questionnaire for adults was synthesized based on five factors including preparation and cooking, production, selection, intake, and disposal. This helped identify abilities such as functional, interactive, and critical literacy. Our study is in line with previous conceptualizations in maintaining a focus on food which broader and more appropriate concept rather than being explicitly nutrition centric. This was further supported by the results of the main survey showing significant positive correlations between food literacy scores and FN- and NQ-scores. The final 25-item food literacy measurement tool comprehensively assesses all areas of food literacy and can identify vulnerable groups based on the scores for each factor of this measurement tool. It was reported that this can be a reliable support in planning and evaluating precise educational strategies [34]. NQ-score is a simple dietary evaluation tool that has been surveyed and validated nationwide in Korean adults. This is the first result showing that food literacy scores are significantly associated with the NQ-score in Korean adults, demonstrating the role of this food literacy measurement tool. 

Interestingly, the EFA showed that the factors of the food system dimension, not the literacy dimension, were clearly distinguishable. Further, some items were reclassified into more suitable areas than their previously existing theoretical classification through EFA. This reclassification via EFA and the results of the focus group interview showed that the food system as perceived by the general public in their role as consumers may differ slightly from the theoretical definition. For example, questions on ingredients and nutrition labels that were previously categorized in the selection domain were reclassified to the production factor. To the consumer, these aspects may be considered information at the production domain for processed foods, which are as important as fresh produce in modern society. Elements related to the distribution method were included in the selection factor, possibly because from the consumers’ viewpoint, the distribution method greatly impacts product choice.

The limitation of this study includes that the number of survey participants was small and limited to adults living in Korea. To overcome this limitation, the gender, age, education, and income level of the study participants were equally distributed. Additionally, to reflect various geographic characteristics, research participants were recruited to have a pattern similar to the regional population ratio [26], and 21% of the included respondents were rural residents. Further, it should be acknowledged that this study used convenience sampling in the validation of the final questionnaire. Additional studies considering concurrent validity, discriminant validity, and invariance across cultures and countries would be needed. Assignment of weights to particular domains may be necessary according to the purpose of study in the future. Collectively, a validated food literacy measurement tool was developed based on a conceptual framework and validated for practical use. This can expand and integrate the concept of food literacy and food systems toward sustainability of diet. Further study on food literacy measurements for children, adolescents, and older adults that considers the characteristics of each life stage is needed.

## 5. Conclusions

In conclusion, we propose an integrated conceptual framework for food literacy that reflects the food system as well as concepts in the previous literature. Furthermore, the food literacy measurement tool we developed has proven validity and reliability in both expert and general populations, reflecting various demographic characteristics. This tool can contribute to solving various food-related problems by more accurately understanding adults’ food literacy levels and establishing effective improvement strategies.

## Figures and Tables

**Table 1:** Question list for focus group interview.

Stage	Question
Starting question	• Please introduce yourself briefly.
Introductory question	• How interested are you in food?
Conversion question	• Have you ever used food-related information?
Main questions	Please describe the various types of food information. Please describe your experience in applying food information. Please explain the possible results of improving “food literacy” that are related to this food information. Please explain the meaning of “food literacy.”
Final questions	• Is there a topic you would like to talk about? • Did the moderator summarize the discussion?

**Table 2:** Conceptual domains of food literacy from previous studies.

Author	Year	Domain
Vidgen and Gallegos	2014 [5]	• Plan and manage
		• Select
		• Prepare
		• Intake
Cullen, et al.	2015 [12]	• Individual food skills (knowledge, access, values, beliefs, culture)
		• Community food security (local food system, programs, availability, etc.)
Perry, et al.	2017 [13]	• Food and nutrition knowledge
		• Food skills
		• Self-efficacy and confidence
		• Ecologic (beyond self)
		• Food decisions
Krause, et al.	2018 [14]	• Functional literacy
		• Interactive literacy
		• Critical literacy
Slater, et al.	2018 [28]	• Functional competencies
		• Relational competencies
		• Systems competencies
Thomas, et al.	2019 [29]	• Food and nutrition knowledge
		• Food skills
		• Self-efficacy and confidence
		• Ecologic (beyond self)
		• Food decisions

**Table 3:** Categorization of food literacy components of the food system dimension derived from focus group interviews.

Domain	Element
Production	Country and region of origin Agricultural food certification (organic, pesticide free, etc.) Genetically modified organisms
Processing	Packaging, packaged amount Food components, ingredients
Distribution	Shelf life Transportation methods (local food, farm-to-home delivery)
Selection	Name of brand/product, advertisement Quality (freshness), quality/price ratio, price and time Taste Traditions and culture (holyday, meetings) Ethical consumption (fair trade, animal welfare)
Preparation and cooking	Recipes Seasoning Preparation and storage of ingredients Hygiene
Intake	Health Nutrients
Disposal	Food waste Recycling Waste reduction (use of reusable shopping bags)

**Table 4:** Demographic characteristics of the participants in the main survey.

Variables	Total (*n* = 200)	Men (*n* = 100)	Women (*n* = 100)	*p*-Value
Age				
19–39	110 (55.0%)	55 (55.0%)	55 (55.0%)	1.00
40–64	90 (45.0%)	45 (45.0%)	45 (45.0%)	
**Education**				
≤Middle school	51 (25.5%)	25 (25.0%)	26 (26.0%)	0.66
High school	125 (62.5%)	64 (64.0%)	61 (61.0%)	
≥College	24 (12.0%)	11 (11.0%)	13 (13.0%)	
**Household income**				
KRW < 2,000,000	39 (19.5%)	19 (19.0%)	20 (20.0%)	0.97
KRW 2~4,000,000	47 (23.5%)	25 (25.0%)	22 (22.0%)	
KRW 4~6,000,000	51 (25.5%)	25 (25.0%)	26 (26.0%)	
KRW > 6,000,000	63 (31.5%)	31 (31.0%)	32 (32.0%)	
**Region**				
Seoul capital area	102 (51.0%)	49 (49.0%)	53 (53.0%)	0.58
Jeolla-do	21 (10.5%)	14 (14.0%)	7 (7.0%)	
Gangwon-do	9 (4.5%)	4 (4.0%)	5 (5.0%)	
Chungcheong-do	21 (10.5%)	11 (11.0%)	10 (10.0%)	
Gyeongsang-do	47 (23.5%)	22 (22.0%)	25 (25.0%)	
**Residential area**				
Urban	158 (79.0%)	75 (75.0%)	83 (83.0%)	0.16
Rural	42 (21.0%)	25 (25.0%)	17 (17.0%)	

*n* (%) Numbers in brackets present percentage of participants. KRW, Kores Won.

**Table 5:** Results of the exploratory factor analysis on the final questionnaire (*n* = 200).

Factor	Item	Communalities	Factor Loading				
			1	2	3	4	5
Preparation and cooking	I can explain the pros and cons of my usual diet. ^a2^	0.57	0.64	0.12	0.11	0.08	0.35
	I usually cook and store food with care, as I am cautious of food poisoning. ^c3^	0.57	0.64	0.24	0.10	0.22	0.22
	I usually check the shelf life of food. ^a4^	0.45	0.63	0.21	0.02	0.11	−0.04
	I try to get accurate information about food and health. ^a4^	0.65	0.62	0.35	0.19	0.22	0.23
	I can determine the condition of food hygiene by watching the meal preparation and cooking process. ^c4^	0.50	0.61	0.11	0.24	0.12	0.22
	I store food in a way that maintains food quality. ^a5^	0.56	0.58	0.02	0.15	0.34	0.29
	I can talk about the pros and cons of Korean food culture. ^c5^	0.55	0.57	0.19	0.41	0.06	0.10
Production	I usually check for the genetically modified organism label on food products. ^a1^	0.66	0.09	0.78	−0.06	0.13	0.16
	I usually check for the agricultural food certification (organic, pesticide free, etc.) label. ^a1^	0.72	0.31	0.77	0.11	0.16	0.04
	I usually check the ingredients in processed foods, such as food content, food additives, etc. ^a1^	0.67	0.17	0.72	0.18	0.13	0.28
	I can find information about food production, such as the “animal welfare” certification for meat and eggs. ^b1^	0.62	0.06	0.67	0.41	−0.02	0.04
	I usually check for the food’s country of origin. ^c2^	0.67	0.49	0.63	0.12	0.06	0.12
	I usually select food based on nutrition labels. ^a3^	0.58	0.14	0.59	0.17	0.08	0.42
	I am aware of how different food transportation methods impact the environment and society. ^a3^	0.55	0.17	0.53	0.37	0.29	−0.12
Selection	If I have any questions about food issues, I can find accurate information from experts or reliable organizations. ^b2^	0.65	0.01	0.09	0.77	0.14	0.17
	I can find information on the various distribution methods, such as local food. ^a3^	0.68	0.30	0.26	0.72	0.06	−0.07
	I can buy food in an efficient way that saves money, time, etc. ^b3^	0.50	0.10	0.20	0.54	0.13	0.38
	I can look up or ask about various ways to judge the quality (taste, freshness, etc.) of food. ^c3^	0.56	0.34	0.21	0.53	0.14	0.30
	I can determine whether food is necessary for me by watching/reading food advertisements. ^b5^	0.41	0.37	−0.01	0.45	0.20	0.18
Disposal	I usually try to reduce food waste. ^a6^	0.74	0.06	0.09	0.10	0.82	0.22
	I am aware of the environmental impact of food waste and take care when disposing of it. ^b6^	0.70	0.28	0.26	0.11	0.73	0.10
	I can find out the exact methods for recycling food packaging and food waste. ^c6^	0.67	0.37	0.10	0.32	0.65	−0.06
Intake	I can prepare nutritionally balanced meals. ^b3^	0.65	0.27	0.17	0.12	0.12	0.72
	I can find food or a menu that suits my health and circumstances (time, place, costs, etc.). ^a4^	0.67	0.34	0.16	0.36	0.06	0.63
	I usually try to eat a variety of food groups, including grains, fish, meat, vegetables, fruits, milk, etc. ^a5^	0.50	0.27	0.24	0.06	0.38	0.47
	Eigenvalue	-	9.37	1.89	1.44	1.28	1.05
	Cumulative% of variance	-	15.50	30.86	42.26	51.41	60.10

^a,b,c^ Domain of literacy dimension (^a^: functional literacy, ^b^: interactive literacy, ^c^: critical literacy); ^1~6^ Domain of food system dimension (^1^: production, ^2^: distribution, ^3^: selection, ^4^: preparation and cooking, ^5^: intake, ^6^: disposal).

**Table 6:** Associations of food literacy scores with food knowledge and nutrition quotient scores (N = 200).

Variables	Model 1					Model 2				
	Coef.	SE	*p*-Value	95% CI		Coef.	SE	*p*-Value	95% CI	
Food knowledge score										
Food literacy score	0.028	0.006	<0.001	0.017	0.039	0.030	0.006	<0.001	0.018	0.041
Nutrition quotient score										
Food literacy score	0.351	0.038	<0.001	0.275	0.426	0.312	0.039	<0.001	0.235	0.389

Coef. = coefficient; CI = confidential interval; SE = standard error. Model 1: crude model, Model 2: adjusted for age, gender, and household income level.

## Supplementary Materials

The following are available online at https://www.mdpi.com/2072-6643/12/11/3300/s1, Table S1: Lists of literature including questions related to food literacy.

## References

[1] GBD 2017 Diet Collaborators (2019). Health effects of dietary risks in 195 countries, 1990–2017: A systematic analysis for the Global Burden of Disease Study 2017. Lancet.

[2] United Nations, Sustainable Development Goals. https://sustainabledevelopment.un.org/sdgs.

[3] Lang T. (2003). Food industrialisation and food power: Implications for food governance. Dev. Policy Rev..

[4] Foresight Obesity System Influence Diagram. https://assets.publishing.service.gov.uk/government/uploads/system/uploads/attachment_data/file/296290/obesity-map-full-hi-res.pdf.

[5] Vidgen H.A., Gallegos D. (2014). Defining food literacy and its components. Appetite.

[6] Neuhauser L., Rothschild R., Rodríguez F.M. (2007). MyPyramid.gov: Assessment of literacy, cultural and linguistic factors in the usda food pyramid web site. J. Nutr. Educ. Behav..

[7] Zoellner J., You W., Connell C., Smith-Ray R.L., Allen K., Tucker K.L., Davy B.M., Estabrooks P. (2011). Health literacy is associated with healthy eating index scores and sugar-sweetened beverage intake: Findings from the rural Lower Mississippi Delta. J. Am. Diet. Assoc..

[8] Persoskie A., Hennessy E., Nelson W.L. (2017). US Consumers’ Understanding of nutrition labels in 2013: The importance of health literacy. Prev. Chronic Dis..

[9] Majchrzak M., Shannon K., Pleasant A., Cabe J., Rella E., Giboyeaux K., McCollum G., Thomson C. (2016). Using the power of culinary nutrition, health literacy, and Partnerships to prevent and reverse chronic disease in low-income communities. J. Acad. Nutr. Diet..

[10] Rothman R.L., Housam R., Weiss H., Davis D., Gregory R., Gebretsadik T., Shintani A., Elasy T.A. (2006). Patient Understanding of Food Labels: The Role of Literacy and Numeracy. Am. J. Prev. Med..

[11] Cullerton K., Vidgen H.A., Gallegos D. (2012). A Review of Food Literacy Interventions Targeting Disadvantaged Young People.

[12] Cullen T., Hatch J., Martin W., Higgins J.W. (2015). Food literacy: Definition and framework for action. Can. J. Diet. Pract. Res..

[13] Azevedo Perry E., Thomas H., Samra H.R., Edmonstone S., Davidson L., Faulkner A., Petermann L., Manafò E., Kirkpatrick S.I. (2017). Identifying attributes of food literacy: A scoping review. Public Health Nutr..

[14] Krause C., Sommerhalder K., Beer-Borst S., Abel T. (2018). Just a subtle difference? Findings from a systematic review on definitions of nutrition literacy and food literacy. Health Promot. Int..

[15] Yuen E.Y.N., Thomson M., Gardiner H. (2018). Measuring nutrition and food literacy in adults: A systematic review and appraisal of existing measurement tools. Health Lit. Res. Pract..

[16] Choe S.J., Ji S.M., Paik H.Y., Hong S.M. (2003). A study on the eating habits and dietary consciousness of adults in urban area. J. Korean Soc. Food Sci. Nutr..

[17] Kim Y., Seo S., Kwon O., Cho M.S. (2012). Comparisons of dietary behavior, food intake, and satisfaction with food-related life between the elderly living in urban and rural areas. Korean J. Nutr..

[18] Krueger R.A., Mary A.C. (2009). Focus Groups: A Practical Guide for Applied Research.

[19] Shin G.R., Jo M.O., Yang J.H. (2004). Qualitative Research Methodology.

[20] Cho Y.H. (1999). Qualitative Research.

[21] Lawshe C.H. (1975). A quantitative approach to content validity. Pers. Psychol..

[22] Lynn M.R. (1986). Determination and quantification of content validity. Nurs. Res..

[23] Lee J.S., Kim H.Y., Hwang J.Y., Kwon S., Chung H.R., Kwak T.K., Kang M.H., Choi Y.S. (2018). Development of Nutrition Quotient for Korean adults: Item selection and validation of factor structure*. J. Nutr. Health.

[24] Guadagnoli E., Velicer W.F. (1988). Relation of sample size to the stability of component patterns. Psychol. Bull..

[25] Kyriazos T.A. (2018). Applied psychometrics: Sample size and sample power considerations in factor analysis (EFA, CFA) and SEM in general. Psychology.

[26] Korean Statistical Information Service (2019). Population Census.

[27] Nunally J.C., Bernstein I.H. (1978). Psychometric Theory.

[28] Slater J., Falkenberg T., Rutherford J., Colatruglio S. (2018). Food literacy competencies: A conceptual framework for youth transitioning to adulthood. Int. J. Consum. Stud..

[29] Thomas H., Azevedo Perry E., Slack J., Samra H.R., Manowiec E., Petermann L., Manafò E., Kirkpatrick S.I. (2019). Complexities in conceptualizing and measuring food literacy. J. Acad. Nutr. Diet..

[30] Wilkins J., Eames-Sheavly M. (2011). A Primer on Community Food Systems: Linking Food, Nutrition and Agriculture.

[31] Rutter M. (2012). Resilience as a dynamic concept. Dev. Psychopathol..

[32] Truman E., Lane D., Elliott C. (2017). Defining food literacy: A scoping review. Appetite.

[33] Nguyen H. (2018). Sustainable Food Systems Concept and Framework.

[34] Wickham C.A., Carbone E.T. (2018). What’s technology cooking up? A systematic review of the use of technology in adolescent food literacy programs. Appetite.

