# Supplementary material for: Development of a Comprehensive Food Literacy Measurement Tool Integrating the Food System and Sustainability
Source: Nutrients. 2020 Oct 28;12(11):3300. doi: 10.3390/nu12113300 (PMC7692683; doi:10.3390/nu12113300)
Supplement: Supplementary file 1 [file nutrients-12-03300-s001.pdf]

Supplementary Table 1. Lists of literature including questions related to food literacy

	Authors	Year	Number of questions	Reference
1	Vidgen, H. & Gallegos, D	2013	75	What is Food Literacy and Does It Influence What We Eat?
2	Poelman, M.P. et al.	2018	29	Towards the Measurement of Food Literacy with Respect to Healthy Eating: The Development and Validation of the Self Perceived Food Literacy Scale Among an Adult Sample in the Netherlands
3	Gréa, K.C et al.	2018	12	A Short Food Literacy Questionnaire (SFLQ) for Adults: Findings from A Swiss Validation Study
4	Begley, A et al.	2019	6	Identifying Participants Who Would Benefit the Most from an Adult Food-Literacy Program
5	Amin, S.A. et al.	2018	6	Identifying Food Literacy Educational Opportunities for Youth
6	Garcia-Gonzalez, A. et al.	2018	9	Identifying Factors Related to Food Agency: Cooking Habits in the Spanish Adult Population-A Cross-Sectional Study
7	De Souza, R.S. et al.	2015	4	General Nutrition Knowledge Questionnaire - Modified and Validated for Use in German Adolescent Athletes
8	Fordyce, V.S.	2018	22	A Food Literacy Model for Food Education Program Design and Evaluation
9	Begley, A. et al.	2019	20	Examining the Association Between Food Literacy and Food Insecurity
10	Begley, A. et al.	2019	4	Effectiveness of an Adult Food Literacy Program
11	Vidgen, H et al.	2014	11	Defining Food Literacy and Its Components
12	Ronto, R. et al.	2016	22	Adolescents' Perspectives on Food Literacy and Its Impact on Their Dietary Behaviours
13	Kim, H.S. & Kim, J.W.	2018	32	The Effects of Dietary Life Education Program for Prospective Elementary Teacher
14	Naigaga, D.A. et al.	2018	5	Assessing Adolescents' Perceived Proficiency in Critically Evaluating Nutrition Information

15	Gibbs, H.D. et al.	2016	5	Assessing the Nutrition Literacy of Parents and Its Relationship with Child Diet Quality
16	Aihara, Y. & Minai, J.	2011	9	Barriers and Catalysts of Nutrition Literacy among Elderly Japanese People
17	Law, Q.P.S. et al.	2019	11	Chinese Adults' Nutrition Label Literacy in Hong Kong: Implications for Nurses
18	Mahmudiono, T. et al.	2018	21	Comparison of Maternal Nutrition Literacy, Dietary Diversity, and Food Security Among Households with and Without Double Burden of Malnutrition in Surabaya, Indonesia
19	Liao, L.L. & Lai, I.J.	2017	32	Construction of Nutrition Literacy Indicators for College Students in Taiwan: A Delphi Consensus Study
20	Doustmohammadian A. et al.	2017	51	Developing and Validating A Scale to Measure Food and Nutrition Literacy (FNLit) in Elementary School Children in Iran
21	Michou M. et al.	2019	25	Development & Validation of the Greek Version of the Nutrition Literacy Scale
22	Coffman, M.J. & La-Rocque, S.	2012	26	Development and Testing of the Spanish Nutrition Literacy Scale
23	Lio, L.L. et al.	2018	29	Development and Validation of the Nutrition Literacy Measure for Taiwanese College Students
24	Ringland, E.M. et al.	2016	16	Evaluation of an Electronic Tool to Assess Food Label Literacy in Adult Australians: A Pilot Study
25	Ballance, D. & Webb, N.	2015	26	For the Mouths of Babes: Nutrition Literacy Outreach to A Child Care Center
26	Gibbs, H.D. et al.	2016	6	Measuring Nutrition Literacy in Breast Cancer Patients: Development of A Novel Instrument
27	Gibbs, H.D. et al.	2018	4	The Nutrition Literacy Assessment Instrument is A Valid and Reliable Measure of Nutrition Literacy in Adults with Chronic Disease
28	Velardo, S. & Drummond, M.	2019	23	Qualitative Insight into Primary School Children's Nutrition Literacy
29	Gibbs, H.D. et al.	2020	6	Validity of an Updated Nutrition Literacy Assessment Instrument with the New Nutrition Facts Panel
